# Thromboendarterectomy in a patient with Systemic Lupus and antiphospholipid Syndrome, lessons learned from a complex disease interaction

**DOI:** 10.21542/gcsp.2020.15

**Published:** 2020-04-30

**Authors:** Ahmed A. Elmogy, Michael Gergis, Ahmed Hassan, Irini S. Hanna, Magdi Yacoub

**Affiliations:** 1 Aswan Heart Center, Aswan, Egypt; 2 Department of Cardiology, School of Medicine, Cairo University, Cairo, Egypt

**Keywords:** Chronic thromboembolic pulmonary hypertension, Systemic lupus erythematosus, Antiphospholipid syndrome, Pulmonary endarterectomy

## Abstract

Systemic lupus erythematosus (SLE) is the prototypic multisystem autoimmune disorder with a broad spectrum of clinical presentations encompassing almost all organs and tissues^1^. Antiphospholipid syndrome (APS) is an autoimmune disease characterized by the occurrence of venous and/or arterial thrombosis and pregnancy morbidity in the presence of pathogenic autoantibodies known as antiphospholipid antibodies (aPL)^2^.

Chronic thromboembolism is one of the well-known established pathogenesis of pulmonary hypertension, known as chronic thromboembolic pulmonary hypertension (CTEPH)^3^.

APS may be also associated with other diseases, mainly systemic lupus erythematosus (SLE). The presence of secondary APS in SLE patients further aggravate the condition due to recurrent venous thromboembolic showers to the pulmonary vasculature. Pulmonary endarterectomy (PEA) is the treatment of choice for CTEPH with lifelong anticoagulation^4^.

We herein report a rare cause of CTEPH in a 42-year-old Egyptian man who presented with dyspnea WHO-FC III. The patient was diagnosed as a case of CTEPH due to secondary APS. He underwent PEA and was discharged on lifelong anticoagulation. Clinical follow-ups thereafter showed improvement of functional capacity and pulmonary artery pressures. In conclusion, management of such cases was combination of standard treatment of CTEPH, in addition to specific management of secondary APS to avoid recurrence of the disease.

## Introduction

The American College of Rheumatology (ACR) classification criteria were developed for SLE to ensure that cases reported in the literature do in fact have the disease. All features included in the classification criteria are contributing equally without any weight based upon sensitivity and specificity for each individual criterion^1^.

Because SLE is a disease whose course is typified by periodic involvement of one organ system after another, it is apparent that patients must have the disease for years before they fulfill the classification criteria. The presence or absence of SLE might modify the clinical or serological expression of APS. Apart from the classical manifestations, APS patients with associated SLE more frequently display a clinical profile with arthralgia, arthritis, autoimmune hemolytic anemia, livedo reticularis, epilepsy, glomerular thrombosis, myocardial infarction and CTEPH secondary to recurrent deep venous thrombosis (DVT)^5^.

CTEPH is a disease of obstructive pulmonary artery remodeling as a consequence of pulmonary vessels thromboembolism. The diagnosis of CTEPH is based on findings obtained after at least 3 months of effective anticoagulation in order to discriminate this condition from ‘subacute’ PE. These findings are mean pulmonary artery pressure (mPAP) ≥ 25 mmHg with pulmonary artery wedge pressure (PAWP) ≤ 15 mmHg^6^.

Specific diagnostic signs for CTEPH are seen by CT angiography, MR imaging or conventional pulmonary cineangiography, such as ring-like stenosis, webs/slits and chronic total occlusions (pouch lesions or tapered lesions). CT pulmonary angiography (CTPA) has become an established imaging modality for confirming CTEPH. However, this investigation alone cannot exclude the disease. A ventilation/perfusion(V/Q) lung scan has been the screening method of choice in CTEPH because of its higher sensitivity compared with CTPA especially in inexperienced centers^7^. MR imaging of the pulmonary vasculature is still considered inferior to CT but may be complimentary and used according to local experience and practice^8^.

Right heart catheterization (RHC) is an essential diagnostic tool. Preoperative and immediate postoperative PVR is a long-term predictor of prognosis. The final step in the diagnostic pathway is selective pulmonary angiography illustrating ring-like stenosis, webs (‘slits’), pouches, wall irregularities, complete vascular obstructions as well as bronchial collaterals, and supports the technical assessment of operability^6^.

PEA is the treatment of choice for CTEPH. In contrast to surgical embolectomy for acute PE, treatment of CTEPH necessitates a true bilateral endarterectomy through the medial layer of the pulmonary arteries, which is performed under deep hypothermia and circulatory arrest^9^.

Operability of patients with CTEPH is determined by multiple factors that cannot easily be standardized; these are related to the suitability of the patient, the expertise of the surgical team and available resources^6^. Optimal medical treatment for CTEPH consists of anticoagulants and diuretics, and oxygen in cases of heart failure or hypoxemia. Lifelong anticoagulation is recommended, even after PEA. Patients with persistent or recurrent PH after PEA may also be candidates for targeted medical therapy^10^.

The use of targeted therapy in operable patients with severe hemodynamic compromise as a bridge to PEA has not yet been supported by scientific evidence. After PEA, patients should be followed in CTEPH centers, with at least one hemodynamic assessment to be considered at 6–12 months after the intervention^5^. We here describe a patient who underwent pulmonary Thromboendarterectomy for CTEPH for this rare combination and review the complex pathophysiology involved, and the management of these neglected diseases.

### Timeline

**Table:** 

2013	Right lower limb swelling improved spontaneously.
	Left lower limb swelling diagnosed as DVT and was prescribed warfarin for 6 months.
2016	Developed hemoptysis and was diagnosed to be acute PE.
	Diagnosed as SLE with antiphospholipid syndrome.
	Received immunosuppressive and warfarin.
2018	Developed gradually progressive dyspnea occurring on minimal effort, palpitations, and bilateral lower limb edema.
1 month before admission	Developed severe decompensated heart failure and was admitted to Aswan Heart Center.
During hospital stay	Pulmonary endarterectomy and tricuspid valve repair was performed. The patient had smooth post-operative recovery and was discharged on medical therapy

### Case report

**Figure 1.**
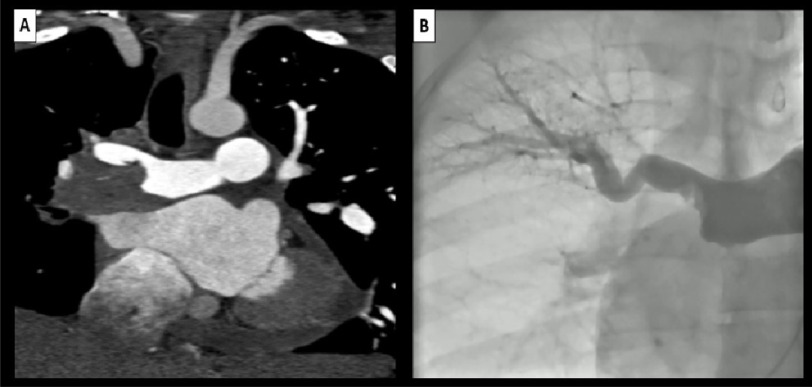
(A) CTPA is showing large flling defect in the distal right pulmonary artery causing subtotal occlusion of the right upper lobar branch and total occlusion of the middle and lower right lobar branches with free fow of contrast through the main and lef pulmonary arteries. (B) Selective pulmonary angiography is showing flling defect in the distal right pulmonary artery with subtotal occlusion of the right upper lobar branch and amputation of middle and lower right lobar branches. CTPA, computed tomography pulmonary angiography.

**Figure 2.**
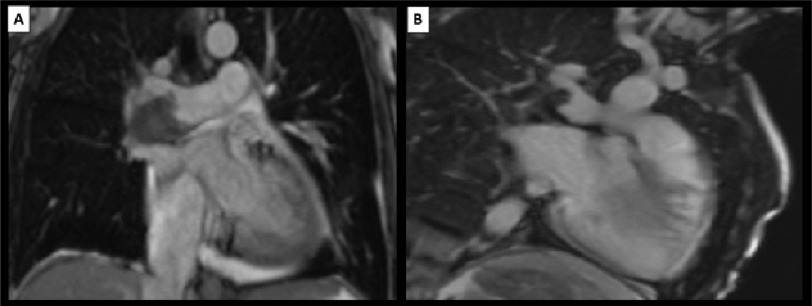
(A) Localizer sequence of cardiac MRI is showing low signal area in the same corresponding sites as detected by CTPA. (B) Early phase of gadolinium injection in cardiac MRI showing large flling defect in the corresponding sites mentioned earlier in CTPA. MRI, magnetic resonance imaging.

**Figure 3.**
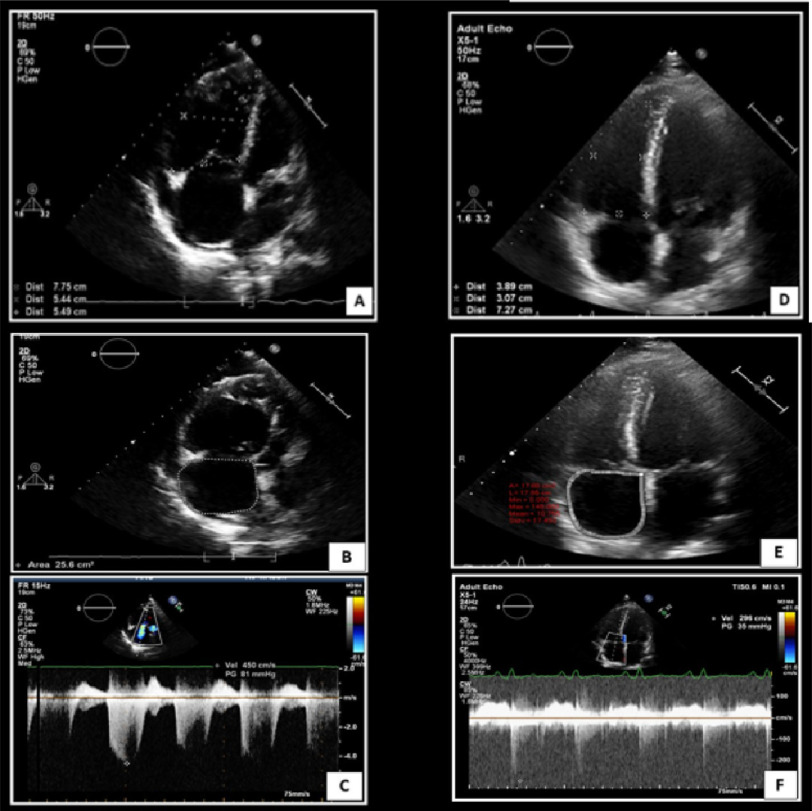
Transthoracic echocardiography apical 4 chamber view, comparing preoperative study (A, B, C) and 6 months follow up study (D, E, F) showing regression of right ventricular dimensions (D), right atrial area (E) and tricuspid regurgitation peak velocity (F) in comparison to previous parameters at baseline study.

**Figure 4.**
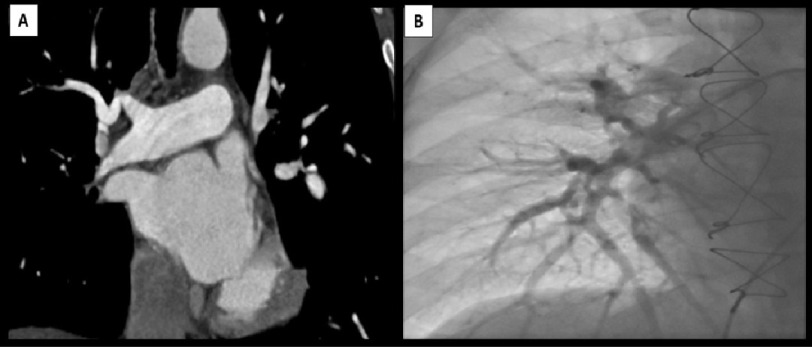
(A) Follow up computed tomography pulmonary angiography showing Complete recanalization the RPA and its resting branches. (B) Follow up Selective pulmonary angiography is showing recanalization of RPA and its branches. RPA, right pulmonary artery.

**Figure 5.**
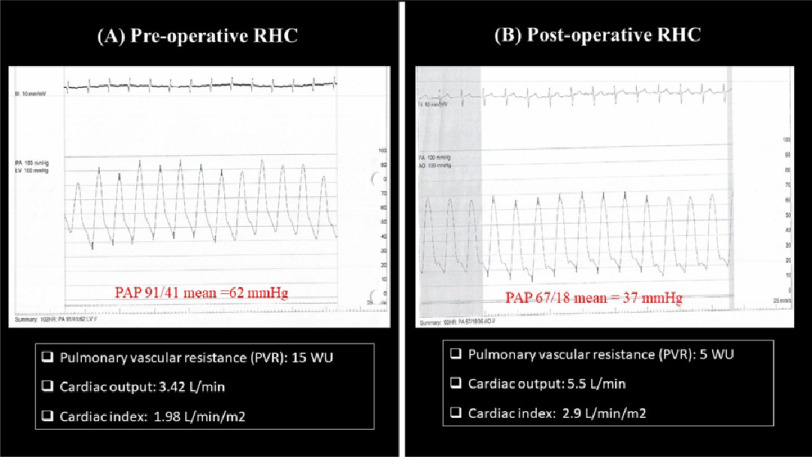
(A) Preoperative RHC, (B) postoperative (six months) follow up RHC showing improvement of hemodynamic parameters in terms of decreased pulmonary artery pressure and pulmonary vascular resistance along with increase of COP and CI. RHC, right heart catheterization; COP, cardiac output; CI, cardiac index.

A 42-year-old gentleman, smoker for 10 years. He was complaining of dyspnea WHO- FC III. On presentation, his vital signs showed a heart rate of 100 b.p.m. and a blood pressure of 113/68 mmHg. Cardiac examination revealed increased intensity of pulmonary component of S2 with pansystolic murmur over tricuspid area. Systemic examination revealed increased jugular venous pressure with systolic expansion of the neck veins and bilateral lower limb pitting edema up to the knee. The remainder of the physical examination was unremarkable. He had history of right lower limb swelling that improved spontaneously 7 years ago. This was followed, 6 months later, by left lower limb swelling diagnosed as DVT and warfarin was prescribed for 6 months. Three years later, he presented by acute PE. He was diagnosed as SLE based on malar rash and non-erosive arthritis observed at the time of presentation. Immunosuppressive agents and lifelong warfarin were prescribed. His sister was diagnosed and died of lupus nephritis. Electrocardiogram showed sinus rhythm with P pulmonale, right ventricular hypertrophy with strain pattern and long QTc. Laboratory investigations were done and anti-nuclear, IgM anticardiolipin, lupus anticoagulant antibodies were positive. Brain natriuretic peptide level was 2110 pg/mL. The rest of the routine laboratory investigations were within normal. Echocardiogram revealed high probability criteria for pulmonary hypertension (severe tricuspid regurgitation (TR) with peak tricuspid regurgitation velocity 4.5 m/s, dilated right ventricle (RV) with RV/ LV basal diameter ratio >1.0, TAPSE=1.3, dilated IVC measuring 24 mm with < 50 % collapse with a sniff, dilated main pulmonary artery measuring 26 mm, enlarged right atrial area measuring 26 cm^2^). CTPA was done revealing large filling defect in the distal right pulmonary artery extending to the origin of upper lobar branch causing partial obstruction and causing total obstruction of middle and lower lobar branches with free flow of contrast through main and left pulmonary arteries (Figure 1A). Cardiac MRI revealed dilated impaired RV (EDVI=300 ml/m^2^, EDSI=243 ml/m^2^, EF=19%), flattening of septum during systole (pressure overload), severe TR (regurgitant fraction 59%) with low signal thrombus (Figure 2A), filling defects in early phase of gadolinium injection (Figure 2B) and absence of scar in late gadolinium sequences. RHC confirmed the diagnosis of CTEPH with mPAP of 62 mmHg, PVR=15 Woods unit, LVEDP=9 mmHg and low cardiac Index measuring 1.98 L/min/m^2^. Complementary selective pulmonary angiography confirmed CTPA findings (Figure 1B). The patient underwent Pulmonary endarterectomy and tricuspid valve repair with smooth post-operative recovery. He was discharged on warfarin, hydroxychloroquine, small dose of prednisolone and diuretics. Clinical follow-ups at three months showed symptomatic improvement (WHO- FC I), he achieved 550 meters in 6-minutes walk test (6MWT) in comparison to 370meters preoperatively. Six months follow up transthoracic echocardiography showed regression of right ventricle dimensions in comparison to baseline study, with right atrial area measuring 17.8 cm^2^ and mild tricuspid regurgitation with peak TR velocity 2.4 m/s (Figure 3). CTPA was done and revealed successful revascularization of the previously occluded right middle and lower lobar branches (Figure 4A), this was confirmed by Invasive pulmonary angiography (Figure 4B). Follow up RHC showed drop of mPAP to 37 mmHg, PVR to 5 Woods unit and increased cardiac index to 2.9 L/min/m^2^ in comparison to 62 mmHg, 15 Woods unit and 1.98 L/min/m^2^ respectively in the preoperative RHC (Figure 5).

## Discussion

SLE is an autoimmune disorder characterized by antibodies to nuclear and cytoplasmic antigens, multisystem inflammation, protean clinical manifestations, and a relapsing and remitting course^11^.

More than 90% of cases occur in women, frequently starting at childbearing age. The incidence of lupus has nearly tripled in the last 40 years, mainly due to improved diagnosis of mild disease.^1^ The exact cause is unknown but a combination of genetic, environmental, or infectious factors are likely to play a role. SLE is a chronic inflammatory disease that can affect almost any organ system, although it mainly involves the skin, joints, kidneys, blood cells, and nervous system. Its presentation and course are highly variable, ranging from indolent to fulminant.^1^ The following clinical manifestations are more commonly found than in adults; malar rash, ulcers/mucocutaneous involvement, renal involvement, proteinuria, urinary cellular casts, seizures, thrombocytopenia, hemolytic anemia, fever, lymphadenopathy.

The APS is defined by two major components; the occurrence of at least one clinical feature as vascular event or pregnancy morbidity and the presence of at least one type of autoantibody known as an antiphospholipid antibody (aPL) on two separate occasions at least 12 weeks apart^12^.

Antiphospholipid syndrome can be primary or secondary, connected with other autoimmune diseases, such as SLE. Approximately 40% of patients with SLE, aPL is present, while less than 40% of them have episodes of thrombosis. However, it is estimated that APS may develop in up to 50-70% of aPL-positive SLE patients during 20 years of follow-up. Difficulties in the diagnosis of APS in SLE patients result from the fact, that a number of symptoms typical of primary APS also occurs in the ACR classification criteria for SLE^12^. Thromboses are the most common clinical manifestation of APS. The most common sites for venous thrombosis are deep venous thrombosis in the veins of the calf.

Chronic thromboembolic pulmonary hypertension occurs secondary to recurrent thromboembolic showers from deep venous thrombosis. Hence, secondary APS is the forerunner of recurrent deep venous thrombosis as in this case. The patient was diagnosed to have SLE as at least four criteria were fulfilled; malar rash, non-erosive arthritis, positive ANA, and positive APL antibodies with family history of lupus nephritis. He was diagnosed to be pulmonary hypertensive due to chronic thromboembolism by CTPA and confirmed by RHC according to current ESC guidelines on pulmonary hypertension recommendations. As a result, he underwent the standard of care therapy, PEA, with clinical as well as hemodynamic recovery. Lifelong anticoagulation, hydroxychloroquine and small dose of steroids were prescribed as indicated in his case. Lifelong anticoagulation is indicated in such patients. Current guidelines recommend direct oral anticoagulants (DOACs) over vitamin K antagonist (VKA) for the treatment of VTE in general. On the other hand, the ESC as well as European Medicines Agency (EMA) Pharmacovigilance Risk Assessment Committee stated that DOACs are not recommended for patients with a history of thrombosis who are diagnosed with antiphospholipid syndrome.^13^

In particular for patients that are triple positive (for lupus anticoagulant, anticardiolipin antibodies, and anti–beta 2-glycoprotein I antibodies), treatment with DOACs could be associated with increased rates of recurrent thrombotic events compared with VKA therapy. Yet a subset of CTEPH patients will harbor aPL antibodies (and a smaller subset will have APS). It is not possible at the time of diagnosis of unprovoked VTE to know whether APS is present, as the diagnosis requires repeat testing at over 12 weeks^14^.

## Conclusion

Secondary APL due to SLE is a typical, although uncommon, cause of CTEPH. The treatment of choice is PEA as in most cases of CTEPH when feasible. There is a dilemma regarding the use of lifelong anticoagulation with VKA versus DOACs in this subset of patients. However, guideline mediated therapy for SLE is recommended.

## Consent

Informed consent was obtained from this patient for publication of this case history and associated images in line with COPE recommendations.

## CONFLICTS OF INTEREST

None declared.
